# Links Between Body Dysmorphic Disorder (BDD) With Aesthetic Components of Orthodontic Treatment Need Assessed by Orthodontic Patients or Orthodontists (IOTN‐AC, IOTN‐ACE) and Also With Its Risk Factors: An Epidemiological Cross‐Sectional Study

**DOI:** 10.1002/hsr2.71956

**Published:** 2026-05-08

**Authors:** Mehrnaz Moradinezhad, Ozra Niknam, Majid Aryannejad, Mahsa Shishesaz, Asal Fetrati, Vahid Rakhshan

**Affiliations:** ^1^ Oral and Dental Health Research Center Ahvaz Jundishapur University of Medical sciences Ahvaz Iran; ^2^ Department of Orthodontics, School of Dentistry Ahvaz Jundishapur University of Medical Sciences Ahvaz Iran; ^3^ School of Dentistry Ahvaz Jundishapur University of Medical Sciences Ahvaz Iran; ^4^ Department of Oral and Maxillofacial Surgery, School of Dentistry University of Michigan Ann Arbor Michigan USA; ^5^ Department of Anatomy Azad University of Medical Sciences Tehran Iran

**Keywords:** body dysmorphic disorder, esthetic components of the index of orthodontic treatment need (IOTN‐AC), esthetic components of the index of orthodontic treatment need evaluated by the examiner (IOTN‐ACE), obsessive compulsive disorder, orthodontic treatment need, orthodontics, psychiatric disorders

## Abstract

**Background and Aims:**

Body dysmorphic disorder (BDD) is a type of obsessive‐compulsive disorder that can adversely impact an individual's self‐perception. It may influence both the demand for and results of orthodontic treatment, making it a significant concern. Research on BDD among orthodontic patients is scarce, controversial, and methodologically limited. Associations between BDD with IOTN‐AC and IOTN‐ACE have not been studied at all. Moreover, IOTN‐ACE itself (even regardless of BDD) is not studied except in a very few articles.

**Methods:**

The Yale–Brown Obsessive‐Compulsive Scale modified for Body Dysmorphic Disorder (BDD‐YBOCS) was administered to 529 orthodontic patients aged ≥ 12 (112 males, 417 females) in five clinics of xxxx city, 2023–2024. The diagnosis and severity of BDD were determined using both the first three items (BDD‐3) and all twelve items of the questionnaire (BDD‐All). Orthodontists and also patients evaluated the esthetic aspect of the Index of Orthodontic Treatment Need (IOTN‐AC and IOTN‐ACE). Multiple linear/binary logistic regressions and bivariable analyses were used to determine correlations and risk factors (among age/sex/marital status/education level/rhinoplasty/esthetic treatments/time passed since treatment began) (*α* = 0.05).

**Results:**

Mean BBD‐3 and BDD‐All were 3.28 ± 2.35 and 13.15 ± 6.97, respectively. BDD prevalence was 39.3% (BDD‐3) or 15.1% (BDD‐All). IOTN‐AC/ACE were significantly but weakly correlated with all four measures of BDD. Mean IOTN‐AC and IOTN‐ACE scores were 3.19 ± 2.30 and 3.46 ± 2.30, respectively. IOTN‐AC and IOTN‐ACE were moderately correlated with each other (*p* < 0.05). Age was not correlated with BDD, but it was negatively correlated with IOTN‐AC and IOTN‐ACE (*p* < 0.05). Female sex and lower IOTN‐AC/ACE scores were associated with BDD (regression *p* < 0.05).

**Conclusion:**

BDD was significantly yet weakly associated with IOTN‐AC/IOTN‐ACE. BDD may be more prevalent in women. Rhinoplasty needs further research. Other factors might not be risk factors.

## 1 Introduction

Demand rather than need may have a greater effect on orthodontic decision‐making [1]. Besides functional improvements, orthodontists should also consider patients' aesthetic satisfaction [1, 2]. Thus, orthodontics and psychology go hand‐in‐hand [3]. Various factors such as self‐confidence, work success, quality of life, emotional well‐being, social relationships, and work success are all significantly influenced by appearance and oral health [4, 5, 6, 7]. In recent years, patients have sought orthodontic treatment to enhance their appearance and quality of life [4, 5, 6, 8], as it boosts self‐esteem and lessens social anxiety [9, 10]. As a result, body image is becoming increasingly important in orthodontics [8].

Esthetic expectations and body image may sometimes be severely unrealistic; there is a psychiatric disorder called body dysmorphic disorder (BDD), also called body dysmorphia, dysmorphophobia, or dysmorphic syndrome, which is a form of obsessive‐compulsive disorder (OCD) [11]. BDD is a multifactorial disorder characterized by: (1) preoccupation with defects seen in the patient's physical appearance that are not visible to other people or are small. (2) Repetitive behaviors such as checking oneself in the mirror and putting on too much makeup due to concerns about their appearance. (3) Anxiety or damage to social functioning caused by excessive self‐consciousness. (4) Excessive physical self‐consciousness that cannot be explained by concerns about the weight as in eating disorders [8, 11]. An individual suffering from BDD is excessively concerned about one or more minor defects in their body image and appearance including problems unnoticeable by other people [12, 13, 14]. Many different behaviors or mental actions can be caused by their anxieties about their appearance, such as hiding their perceived imperfections, glancing in the mirror, or comparing oneself to others [15]. Anxiety disorders, major depressive disorder, and obsessive‐compulsive disorder are among the mental conditions that can coexist with BDD [16]. The head and face are the main areas of concern for appearance in the majority of BDD sufferers [17]. Patients frequently seek out cosmetic and non‐psychiatric therapies because they are unaware of the underlying nature of their condition [17]. Dentistry is one of these aesthetic specialties. Dentists, plastic surgeons, maxillofacial surgeons and orthodontists may be the first clinicians who encounter BDD patients [13]. Most BDD patients will seek dentists for braces, dental bleaching, and jaw surgery; in this regard, orthodontic problems may be related to interdental spaces, misalignments of upper and lower midlines, improper tooth sizes, and rotations [8, 18]. The majority of BDD patients who undergo routine dental or orthodontic treatment are not happy with the outcome and often look for alternative dentists and orthodontists [8]. For this reason, the psychological assessment and expectations of patients pursuing orthodontic treatment are a crucial component of a comprehensive evaluation [8].

In the general population, BDD may be present in 0.7%–2.4% of cases [19]. But in other therapeutic contexts, particularly those involving cosmetic procedures, BDD can be more common—for example, 3%–53% in cosmetic surgery settings and 9%–12% in dermatology settings [8, 19, 20]. However, there is scarce information regarding BDD in orthodontic settings because there are just four mostly small and methodically limited studies on BDD in orthodontic patients [8, 13, 17]. Two of these studies were small, examining merely 40 and 270 patients [13, 17]. Furthermore, three of these studies only employed the first three BDD questionnaire items—ignoring the remaining 8 items—to diagnose BDD [8, 17]. According to these four reports, the prevalence of BDD among orthodontic patients is 5.2%–7.5% [8, 13, 17].

No research has been done on possible relationships between the esthetic components of orthodontic treatment need and BDD diagnosis or severity. This is important because treatment need is typically identified by clinicians, while patients' opinions matter considerably as well. Therefore, it is of clinical importance to know potential correlations between self‐perceived esthetic judgments and normative esthetic judgements [3, 21, 22]. In this regard, facial esthetics can be self‐perceived by the patient, as in BDD questionnaire or the aesthetic component of the index of orthodontic treatment need (IOTN‐AC). It can also be evaluated by an expert, as in IOTN‐ACE [3]. Moreover, other areas are under‐researched and controversial: the orthodontic literature has several gaps as well as inadequacies and controversies when it comes to BDD and/or IOTN, especially IOTN‐ACE [22]. Controversies extend beyond orthodontic populations; even in non‐orthodontist populations, risk factors of BDD are controversial [8, 13, 17, 23]. Therefore, this study was conducted for the first time. The absence of any correlations between any of the variables was the null hypothesis.

## 2 Materials and Methods

This epidemiological, multicenter, analytical cross‐sectional study was conducted on orthodontic patients visiting five centers (four private orthodontic offices and the Department of Orthodontics, Ahvaz Jundishapur University of Medical Sciences, Ahvaz, Iran) between October 24, 2023 and December 2024. The protocol and its ethics were approved by the Research Ethics Committee of Ahvaz Jundishapur University of Medical Sciences, Ahvaz, Iran (ethics code: IR.AJUMS.REC.1402.358). The study's goals and methodology were described to all participants (and in the case of underaged children, to their parents). Patients and their parents were guaranteed about the confidentiality of their information. Finally, all patients and/or their parents (in the case of children) signed written informed consent forms. All methods were performed in accordance with the relevant guidelines and regulations (including the Declaration of Helsinki); all experimental protocols were approved by the Institutional Review Board of Ahvaz Jundishapur University of Medical Sciences, Ahvaz, Iran (ethics code: IR.AJUMS.REC.1402.358).

### 2.1 Sample

The inclusion criteria included orthodontic patients 12 years old or older who agreed to join the study. In terms of orthodontic treatment status, the included patients were either in the pre‐treatment stage (before starting any orthodontic treatment) or during orthodontic treatment. The exclusion criteria were a history of previous orthodontic treatment, patients with skeletal abnormalities with ANB < 0 or > 4 degrees, craniofacial syndromes, patients with cleft palate and lip, patients with skeletal malocclusion who needed orthognathic surgery, children under 12 years old, and individuals who could not fill out the questionnaire or were not consent to do so.

### 2.2 Sample Size Calculation

The sample size was calculated based on the findings of a pilot study of 20 participants to obtain high powers above 95%. The correlation between BDD score and IOTN‐AC in the pilot study was calculated to be 0.21. This parameter along with an alpha of 0.05 and a power of 95% were used in the following formula: *n* = $\frac{{\left(Z_{1-\frac{\propto }{2}}+Z_{1-\beta }\right)}^{2}}{{\left(\frac{1}{2}\ln\frac{1+r}{1-r}\right)}^{2}}$ + 3. The resulted sample size was 404 participants. We increased this sample size to 550 participants in order to improve the reliability and power.

### 2.3 Data Collection

#### 2.3.1 Patient Characteristics

Collected were patients' sex, age, marital status, level of education, plus a history of previous orthodontic consultations [8, 18, 22], as well as a history of rhinoplasty, esthetic medical treatments, and non‐esthetic medical treatments were recorded.

#### 2.3.2 IOTN‐AC

The esthetic component of the index of orthodontic treatment need (IOTN‐AC) was determined by the patient by looking at a panel of 10 color photographs that differed in the extent of esthetic, ranging from 1 to 10: grade 1 represents the most beautiful and grade 10 represents the least beautiful smile. The patient selects the image that most resembles his own smile, among the available 10 images.

#### 2.3.3 IOTN‐ACE

The IOTN‐ACE index was similar to IOTN‐AC with the only difference that instead of the patient, this index was filled out by an orthodontist at each center—a total of four different orthodontists performed this.

#### 2.3.4 BDD

A translation of the English questionnaire, which shown great validity and repeatability for participants speaking Persian [24], was utilized in this investigation. Twelve items specifically tailored for body deformity were included in the self‐report version of the Yale–Brown Obsessive‐Compulsive Scale adapted for physical Dysmorphic Disorder (BDD‐YBOCS) questionnaire [25]. The focus of this questionnaire was on obsessive thoughts in questions 1 through 5 and on compulsive actions in questions 6 through 10. Two more questions about avoidance (question 11) and insight (question 12) were also included. The first five questions measure obsessive preoccupations with perceived appearance flaws (control over preoccupations, resistance against preoccupations, interference in functioning and suffering owing to perceived appearance defects, and time preoccupied). Similar to items 1–5 (time spent engaging in the behaviors, interference with functioning as a result of the behaviors, distress if the behaviors are stopped, and resistance of and control over the behaviors), items 6–10 evaluate repetitive behaviors associated with BDD (such as excessive grooming and mirror checking). Item 11 evaluates awareness of appearance beliefs (e.g., “I am ugly”), while item 12 evaluates avoidance (e.g., of social activities or work/school) due to symptoms of BDD. The overall score goes from 0 to 48, with higher values denoting more severe symptoms. The scores for each item range from 0 (no symptoms) to 4 (intense symptoms) [26]. Items 1 through 12 were categorized as follows: (1) time spent thinking, (2) preoccupation‐related interference, (3) preoccupation‐related distress, (4) resistance against preoccupation, (5) control of thoughts, (6) time spent engaging in activities, (7) behavior‐related interference, (8) behavior‐related distress, (9) resistance against behaviors, (10) behavior control, (11) insight, and (12) avoidance [27]. On a Likert scale, respondents marked one of the following statements they agreed with for each question: 0, 1, 2, 3, and 4 [22, 26].

The Yale–Brown BDD‐YBOCS obsessive‐compulsive disorder questionnaire has been shown to be valid and reliable, making it a useful instrument for evaluating symptoms of BDD. According to Phillips et al. [25], the retest type's reliability throughout the 1‐week period was adequate (*r* = 0.88). This scale has a high level of internal consistency, as seen by its Cronbach's alpha coefficient of 0.80 for internal consistency. When compared to the Brief Psychiatric Rating Scale (BPRS), diagnostic validity was adequate. It is consistent with several other psychiatric scales [26]. This measure can also evaluate how well symptoms are improving following therapy. It has been proposed that a score of 20 or above be used to diagnose BDD, even though there is no set cut‐off point [22, 25, 28, 29, 30, 31]. Philips [32] suggest that questions 4–12 center on assessing the intensity of symptoms, whereas the first three questions pertain to the BDD diagnostic criteria. Philips [32] suggested that the sum of the first three questions (also called BDD‐3 in this article) might somehow mirror the sum of all questions; accordingly, the sum of the first three items can have different values: 3 means there is no BDD; 4–5 means there is mild BDD; 6 means there is mild‐to‐moderate BDD; 7 indicates there is moderate BDD; 8 means there is moderate‐to‐severe BDD; 9 means severe BDD; 10 indicates severe to extremely severe BDD; and 11 or 12 indicate extremely severe BDD [8, 22, 25, 32]. In this regard, each sum score of the first three questions (i.e., BDD‐3) may be roughly equal to that value times 4 on all 12 questions (called BDD‐All in this article): in other words, a 5 on the first three questions (BDD‐3) may be roughly equivalent to a 20 on the full version (BDD‐All); a 6 on BDD‐3 to a total 24 on BDD‐All; a 7 on BDD‐3 to a 28 on BDD‐All; an 8 to a total 32; a 9 to a total 36; a 10 to a total 40; an 11 to a total 44; and a 12 (the maximum score) on the first three question (BDD‐3) to a 48 on BDD‐All (the maximum score) [25, 32].

For the purpose of diagnosing and estimating the prevalence of BDD, most studies have merely employed the first three questions of this 3‐item BDD diagnostic method [8, 17]. For this reason, in this study, we employed both approaches to BDD diagnosis (i.e., we used all 12 items as well as only the first three items) [22]. The sum of the scores of the first three questions and also the sum of the scores of all 12 questions were calculated. A total score equal to or above 20 was considered a definite indication of BDD if all questions are considered. A total score equal to or above four was considered a definite indication of BDD if only the first three questions are considered [25, 32].

### 2.4 Internal Consistency, Test‐Retest Reliability, and Intraobserver Agreement

The internal consistency of BDD questionnaire was 86.1% (Cronbach Alpha). For test‐retest reliability, 14 participants filled out the questionnaire after 1 month. Their BDD results showed 77.9% correlation (*p* < 0.001). Their intra‐observer agreements for their ACE scores were assessed using the intraclass correlation, which showed 61.8% agreement (*p* = 0.002, 95% CI = 22.7%–83.8%, two‐way mixed, consistency). The orthodontist's intra‐observer agreement was 96.2% (*p* < 0.001, 95% CI = 90.2%–98.6%, two‐way mixed, consistency). An attempt was made to reduce biases through a 4‐center design, using standardized questionnaires, and using proper exclusion or inclusion criteria.

### 2.5 Statistical Analyses

Descriptive statistics and 95% confidence intervals (95% CIs) were computed for IOTN‐AC, IOTN‐ACE, BDD‐3 (the sum of the first three questions) and BDD‐All (the sum of all 12 questions). The prevalences of BDD diagnosis based on the full BDD questionnaire and its first three items were separately calculated. The data were analyzed using one‐way analysis of variance (ANOVA), independent‐samples *t*‐test, Spearman correlation coefficient, multiple linear regression, and multiple binary logistic regression of SPSS 26 (IBM, Armonk, NY, USA). The level of significance was set at 0.05.

## 3 Results

There was no missing data. More than 600 patients were examined during 2023–2024, of whom 529 completed all questionnaires. Of the included 529 patients (mean age: 22.04 ± 8.62 years old), 417 (78.8%) were female (mean age: 22.02 ± 8.71 years old, range: 12–63) and 112 (21.2%) were male (mean age: 22.13 ± 8.33 years old, range: 12–48). There was no significant difference between average ages of men and women (*t* test, *p* = 0.908).

Of the participants, 420 (79.4%) were single and 109 (20.6%) were married. Of the participants, 237 (44.8%), 254 (48.0%), 16 (3.0%), and 22 (4.2%) were respectively without a high‐school diploma, had diploma only, had MS degree, and had PhD degrees. Of them, 99 (18.7%) had a history of medical treatment; 8 (1.5%) had a history of previous esthetic treatments and/or rhinoplasty. The status of time passed since the beginning of orthodontic treatment in 529 patients was as follows: 65 (12.3%) were in the Pretreatment stage (no treatment performed yet), 217 (41%): 0–6 months of treatment, 89 (16.8%): 6–12 months of treatment, 64 (12.1%): 12–18 months of treatment, and 94 (17.8%): Above 18 months of treatment, and 77 (16.1%) had a previous history of orthodontic consultation.

### 3.1 Esthetic Component of Orthodontic Treatment Need

**Figure 1:**
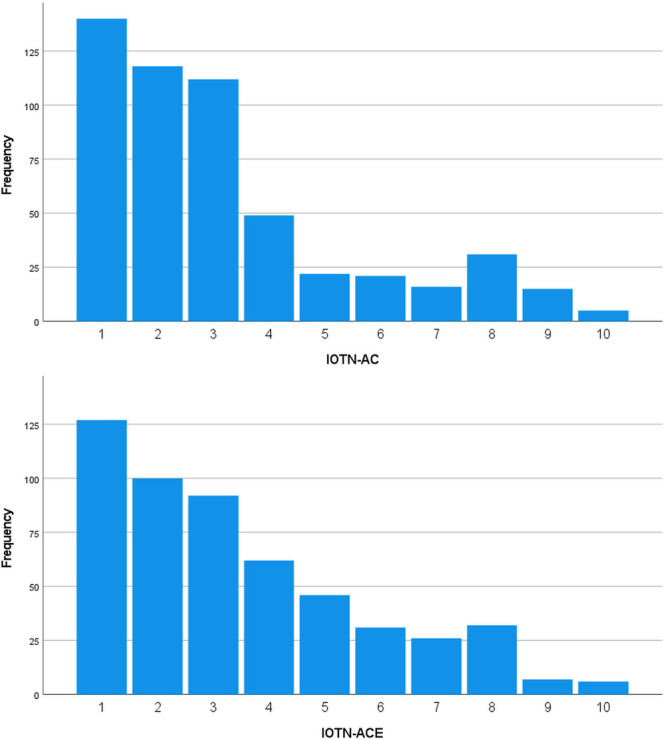
Distributions of IOTN‐AC and IOTN‐ACE scores (*n* = 529). The distributions of milder cases are considerably greater than severer cases.

According to the IOTN‐AC, most patients felt they had a very mild or moderate esthetic treatment need (Figure 1, Table 1). According to the IOTN‐ACE, orthodontists were in agreement with patients (Figure 1, Table 1). There was no difference between men and women in terms of their IOTN‐AC or IOTN‐ACE (Figure 1, Table 1). None of the factors “rhinoplasty history, sex, and education level” were associated with IOTN‐AC or IOTN‐ACE (*t*‐test or ANOVA, Table 1). However, the time passed since beginning of orthodontic treatment was slightly and negatively associated with IOTN‐AC and ACE scores (ANOVA, Table 1). The Tukey test showed four pairwise comparisons for IOTN‐AC and seven pairwise comparisons for IOTN‐ACE (Table 1). Although, IOTN‐AC did not differ between married or single patients, IOTN‐ACE did differ (Table 1): married patients had better smiles than single patients.

**Table 1:** Descriptive statistics and 95% CIs for IOTN‐AC and IOTN‐ACE. The *p* values are calculated using the *t*‐test or one‐way ANOVA.

Variable	IOTN	Group	*N*	Mean	SD	95% CI		Min	Max	*p*
Sex	IOTN‐AC	Female	417	3.22	2.33	2.99	3.44	1	10	0.625
		Male	112	3.10	2.20	2.69	3.51	1	10	
		Total	529	3.19	2.30	3.00	3.39	1	10	
	IOTN‐ACE	Female	417	3.42	2.24	3.20	3.63	1	10	0.460
		Male	112	3.60	2.49	3.13	4.06	1	10	
		Total	529	3.46	2.30	3.26	3.65	1	10	
Education level	IOTN‐AC	Pre‐diploma	237	3.48	2.37	3.18	3.78	1	10	0.059
		Diploma‐BSc	254	3.00	2.25	2.72	3.28	1	10	
		MSc	16	2.81	2.40	1.53	4.09	1	9	
		PhD or higher	22	2.59	1.89	1.75	3.43	1	6	
	IOTN‐ACE	Pre‐diploma	237	3.64	2.29	3.35	3.93	1	10	0.064
		Diploma‐BSc	254	3.39	2.32	3.11	3.68	1	10	
		MSc	16	3.25	2.54	1.89	4.61	1	9	
		PhD or higher	22	2.32	1.49	1.66	2.98	1	6	
Rhinoplasty or esthetic treatments	IOTN‐AC	No	521	3.20	2.30	3.00	3.40	1	10	0.483
		Yes	8	2.63	2.56	0.48	4.77	1	8	
	IOTN‐ACE	No	521	3.47	2.30	3.27	3.67	1	10	0.303
		Yes	8	2.63	1.69	1.22	4.03	1	6	
Marital status	IOTN‐AC	Single	420	3.24	2.28	3.02	3.46	1	10	0.351
		Married	109	3.01	2.38	2.56	3.46	1	10	
	IOTN‐ACE	Single	420	3.60	2.32	3.38	3.83	1	10	0.004
		Married	109	2.89	2.11	2.49	3.29	1	9	
Time passed since beginning orthodontic treatment	IOTN‐AC	Pretreatment	65	3.23	2.34	2.65	3.81	1	10	< 0.001
		0–6 months	217	3.76 ^a,b^	2.39	3.44	4.08	1	10	
		6–12 months	89	3.34 ^c,d^	2.40	2.83	3.84	1	9	
		12–18 months	64	2.31 ^a,c^	1.69	1.89	2.73	1	10	
		Above 18 months	94	2.32 ^b,d^	1.90	1.93	2.71	1	9	
	IOTN‐ACE	Pretreatment	65	4.49 ^e,f,g^	2.51	3.87	5.12	1	10	< 0.001
		0–6 months	217	4.17 ^h,I,j^	2.12	3.89	4.45	1	10	
		6–12 months	89	3.24 ^e,h,k^	2.20	2.77	3.70	1	10	
		12–18 months	64	2.52 ^f,i^	2.20	1.97	3.06	1	10	
		Above 18 months	94	1.94 ^g,j,k^	1.54	1.62	2.25	1	8	

*Note:* Superscript alphabets indicate significant post hoc pairwise comparisons using the Tukey test.

### 3.2 BDD Prevalence and Severity

The distributions of answers to each of the 12 BDD questions are presented in Supporting Information S1: Figure 1. BDD diagnosis and its severity are as follows:

#### 3.2.1 BDD‐3: Based on the Sum of the First 3 BDD Questions

**Figure 2:**
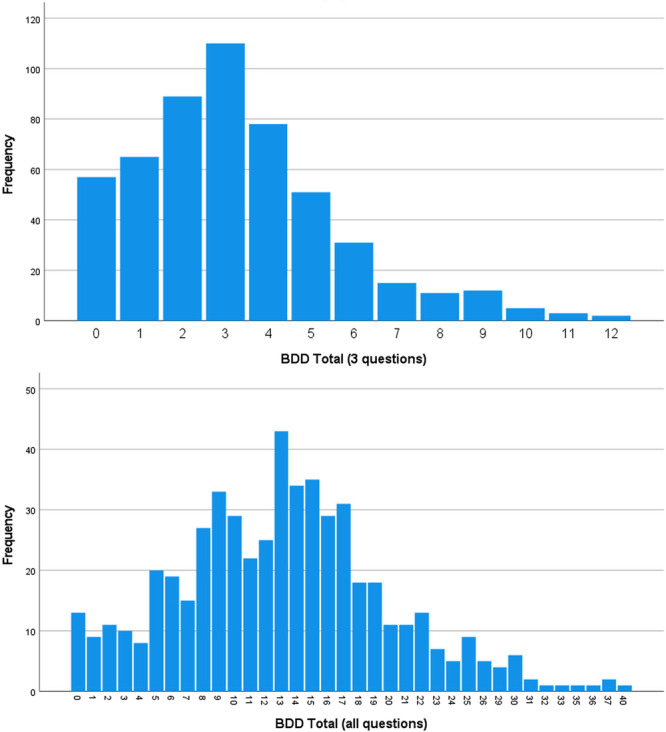
Distributions of BDD‐3 scores (sum of the scores of the first three questions) and BDD‐All scores (sum of scores of all 12 scores, *n* = 529). The distributions of milder cases are greater than severer cases, but cases with moderate total scores are the most frequent.

Of the 529 orthodontic patients, 321 patients had a total BDD‐3 score of 3 or smaller and thus diagnoses as not having BDD. However, 208 patients (39.3%) had total BDD‐3 scores above 3, and therefore, were diagnosed as having BDD (Figure 2). The distribution of severities of BDD were as follows: normal: 321 patients without BDD (60.7%), mild BDD: 129 patients (24.4%), mild‐to‐moderate BDD: 31 patients (5.9%), moderate BDD: 15 patients (2.8%), moderate‐to‐severe BDD: 11 patients (2.1%), severe BDD: 12 patients (2.3%), severe‐to‐extremely severe BDD: 5 patients (0.9%), and extremely severe BDD: 5 patients (0.9%). Females had marginally higher mean BDD‐3 scores than males (Table 2). Mean BDD‐3 scores were not significantly different between levels of other variables (education, rhinoplasty or esthetic treatments, marital status, time passed since beginning the treatment, Table 2).

**Table 2:** Descriptive statistics and 95% CIs for BDD scores based on either the first three questions or all the questions. The *p* values are calculated using the *t*‐test or one‐way ANOVA.

Variable	BDD	Group	*N*	Mean	SD	95% CI		Min	Max	*p*
Sex	BDD‐All (sum of all items)	Female	417	13.46	7.05	12.78	14.14	0	40	0.044
		Male	112	11.97	6.53	10.75	13.20	0	26	
		Both	529	13.15	6.97	12.55	13.74	0	40	
	BDD‐3 (sum of the first 3 items)	Female	417	3.38	2.38	3.15	3.61	0	12	0.057
		Male	112	2.90	2.21	2.49	3.32	0	9	
		Both	529	3.28	2.35	3.08	3.48	0	12	
Education	BDD‐All (sum of all items) items)	Pre‐diploma	237	12.81	7.43	11.86	13.77	0	37	0.655
		Diploma‐BSc	254	13.54	6.54	12.74	14.35	0	40	
		MSc	16	12.44	7.44	8.47	16.40	0	29	
		PhD or higher	22	12.68	6.42	9.83	15.53	2	25	
	BDD‐3 (sum of the first 3 items)	Pre‐diploma	237	3.22	2.55	2.90	3.55	0	12	0.645
		Diploma‐BSc	254	3.38	2.22	3.10	3.65	0	11	
		MSc	16	2.69	1.89	1.68	3.69	0	7	
		PhD or higher	22	3.14	2.01	2.25	4.03	0	8	
Rhinoplasty or esthetic treatments	BDD‐All (all questions)	No	521	13.10	6.94	12.50	13.70	0	40	0.223
		Yes	8	16.13	8.68	8.87	23.38	8	30	
	BDD‐3 (3 questions)	No	521	3.26	2.34	3.06	3.46	0	12	0.139
		Yes	8	4.50	3.16	1.86	7.14	1	9	
Marital status	BDD‐All (all questions)	Single	420	13.17	6.98	12.50	13.84	0	40	0.877
		Married	109	13.06	6.95	11.74	14.37	0	33	
	BDD‐3 (3 questions)	Single	420	3.30	2.34	3.08	3.53	0	12	0.639
		Married	109	3.18	2.42	2.72	3.64	0	11	
Time passed since beginning orthodontic treatment	BDD‐All (sum of all items)	Pretreatment	65	14.68	7.37	12.85	16.50	0	37	
		0–6 months	217	12.77	7.18	11.81	13.73	0	40	0.391
		6–12 months	89	13.27	6.79	11.84	14.70	0	35	
		12–18 months	64	12.66	6.43	11.05	14.26	0	26	
		Above 18 months	94	13.18	6.67	11.81	14.55	0	30	
	BDD‐3 (sum of the first 3 items)	Pretreatment	65	3.69	2.57	3.05	4.33	0	10	0.594
		0–6 months	217	3.21	2.27	2.90	3.51	0	12	
		6–12 months	89	3.36	2.59	2.81	3.90	0	12	
		12–18 months	64	3.22	2.19	2.67	3.76	0	9	
		Above 18 months	94	3.12	2.28	2.65	3.58	0	10	

#### 3.2.2 BDD‐All: Based on the Sum of All 12 BDD Questions

A total of 80 patients had BDD (15.1%, Figure 2). The distribution of severities of BDD were as follows: normal: 449 patients without BDD (84.9%), mild BDD: 42 patients (7.9%), mild‐to‐moderate BDD: 19 patients (3.6%), moderate BDD: 12 patients (2.3%), moderate‐to‐severe BDD: three patients (0.6%), severe BDD: three patients (0.6%), and severe‐to‐extremely severe BDD: 1 patient (0.2%). Females had significantly higher BDD‐All scores compared to males (Table 2). Mean BDD‐All scores were not significantly different between levels of other variables (education, rhinoplasty or esthetic treatments, marital status, time passed since beginning the treatment, Table 2).

### 3.3 Correlations

According to the Spearman correlation coefficient (Table 3), the evaluated four parameters related to BDD (i.e., BDD score sums and BDD diagnosis severities calculated based on BDD score sums) were significantly correlated with each other; the weakest correlations were between the severity of BDD calculated based on all 12 BDD scores (BDD‐All) with either BDD‐3 or BDD‐All. Although the BDD‐3 scores strongly correlated with BDD‐All scores (Table 3), the correlation between binary BBD diagnosis based on the first three questions and all 12 questions was moderate (Spearman Rho = 0.503, *p* < 0.001).

**Table 3:** The results of Spearman correlation coefficient. *N* for each correlation = 529 patients.

		Age	BDD‐All	BDD‐3	BDD severity (BDD‐All)	BDD severity (BDD‐3)	IOTN‐AC	IOTN‐ACE
Age	Rho		0.062	0.030	−0.021	0.005	−0.156	−0.143
	*p*		0.157	0.488	0.633	0.914	< 0.001	0.001
BDD‐All (sum of all questions, continuous)	Rho	0.062		0.827	0.624	0.734	0.145	0.118
	*p*	0.157		< 0.001	< 0.001	< 0.001	0.001	0.007
BDD‐3 (sum of the first three questions, continuous)	Rho	0.030	0.827		0.577	0.882	0.134	0.107
	*p*	0.488	< 0.001		< 0.001	< 0.001	0.002	0.013
BDD severity based on the sum of all 12 questions (BDD‐All, ordinal)	Rho	−0.021	0.624	0.577		0.632	0.142	0.116
	*p*	0.633	< 0.001	< 0.001		< 0.001	0.001	0.007
BDD severity based on the sum of the three first questions (BDD‐3, ordinal)	Rho	0.005	0.734	0.882	0.632		0.128	0.122
	*p*	0.914	< 0.001	< 0.001	< 0.001		0.003	0.005
IOTN‐AC	Rho	−0.156	0.145	0.134	0.142	0.128		0.542
	*p*	< 0.001	0.001	0.002	0.001	0.003		< 0.001
IOTN‐ACE	Rho	−0.143	0.118	0.107	0.116	0.122	0.542	
	*p*	0.001	0.007	0.013	0.007	0.005	< 0.001	

IOTN‐AC and IOTN‐ACE were significantly but weakly correlated with all four measures of BDD, that is, BDD score sums and BDD diagnosis severities calculated based on BDD score sums (Table 3). These two indices (IOTN‐AC and IOTN‐ACE) were moderately correlated with each other (Table 3). Age was not correlated with BDD, but it was negatively correlated with IOTN‐AC and IOTN‐ACE. Nevertheless, this correlation, although statistically significant, was weak (Table 3).

### 3.4 Multivariable Analyses of Factors Influencing BDD‐All Score and BDD Diagnosis

Only two factors were found to be linked with higher full‐12‐item BDD scores by multiple linear regression: female sex and higher IOTN‐AC scores (Table 4, *F* = 2.62, adjusted R‐squared = 0.024, VIF = 1.022 and 2.484, residual's mean square = 47.359). When IOTN‐AC was eliminated, the model likewise found that lower IOTN‐ACE scores were linked to higher BDD scores (Beta = 0.130, *p* = 0.007).

**Table 4:** The results of the multiple linear regression analysis for predictors of BDD‐All (the sum of all 12 questions).

Predictor	*B*	SE	Beta	*t*	*p*	95% CI	
(Constant)	10.496	1.319		7.961	< 0.001	7.906	13.086
Sex	−1.533	0.740	−0.090	−2.070	0.039	−2.987	−0.078
Age	0.002	0.055	0.002	0.035	0.972	−0.106	0.109
Education level	0.454	0.512	0.048	0.887	0.375	−0.551	1.459
Marital status	−0.251	0.988	−0.015	−0.254	0.800	−2.192	1.691
Rhinoplasty or Esthetic Treatments	3.195	2.465	0.056	1.296	0.195	−1.647	8.037
Time passed from the beginning of orthodontic treatment	0.108	0.252	0.020	0.429	0.668	−0.386	0.602
IOTN‐AC	0.384	0.149	0.127	2.587	0.010	0.092	0.676
IOTN‐ACE	0.219	0.159	0.072	1.375	0.170	−0.094	0.532

Abbreviations: β, standardized coefficient; B, unstandardized coefficient; CI, confidence interval; DV, dependent variable; IV, independent variable; SE, standard error for B.

The multiple binary logistic regression analysis (with a −2 Log likelihood, Cox and Snell R‐squared, and Nagelkerke R‐squared respectively equal to 434.984, 0.027, and 0.047) indicated the same variables to be associated with positive BDD diagnosis based on BDD‐All (i.e., scores above 20, Table 5). After removing IOTN‐AC, IOTN‐ACE became significant (*p* = 0.042, OR = 1.0119).

**Table 5:** The results of the multiple binary logistic regression analysis for predictors of BDD diagnosis based on BDD‐All (the sum of all 12 questions).

Predictor	*B*	SE	*p*	OR	95% CI	
Sex	−0.391	0.332	0.238	0.676	0.353	1.295
Age	−0.009	0.024	0.717	0.991	0.946	1.039
Education	0.086	0.217	0.692	1.090	0.712	1.669
Marital status	−0.050	0.419	0.905	0.951	0.419	2.162
Rhinoplasty or esthetic treatments	0.813	0.849	0.338	2.254	0.427	11.897
Time passed from the beginning of orthodontic treatment	−0.028	0.106	0.791	0.972	0.790	1.197
IOTN‐AC	0.139	0.054	0.011	1.149	1.033	1.279
IOTN‐ACE	0.048	0.062	0.437	1.049	0.929	1.185
Constant	−2.227	0.546	< 0.001	0.108		

Abbreviations: B, unstandardized coefficient; CI, confidence interval; SE, standard error for B; OR, odds ratio.

### 3.5 Multivariable Analyses of Factors Influencing BDD‐3 Score and BDD Diagnosis

In terms of BDD‐3 scores, the results were similar to BDD‐All: female sex and higher IOTN‐AC scores (Table 6, *F* = 2.72, adjusted R‐squared = 0.025, VIF = 1.022 and 2.484, residual's mean square = 47.359). When IOTN‐AC was excluded, IOTN‐ACE turned significant (Beta = 0.117, *p* = 0.015).

**Table 6:** The results of the multiple linear regression analysis for predictors of BDD‐3 (the sum of the first 3 questions).

Predictor	*B*	SE	Beta	*t*	*p*	95% CI	
(Constant)	2.550	0.445		5.724	< 0.001	7.906	13.086
Sex	−0.485	0.250	−0.084	−1.939	0.053	−2.987	−0.078
Age	0.003	0.018	0.012	0.182	0.856	−0.106	0.109
Education	0.077	0.173	0.024	0.445	0.656	−0.551	1.459
Marital status	−0.179	0.334	−0.031	−0.535	0.593	−2.192	1.691
Rhinoplasty	1.319	0.833	0.068	1.584	0.114	−1.647	8.037
Time	0.001	0.085	0.000	0.010	0.992	−0.386	0.602
IOTN‐AC	0.142	0.050	0.139	2.825	0.005	0.092	0.676
IOTN‐ACE	0.055	0.054	0.054	1.028	0.305	−0.094	0.532

Abbreviations: β, standardized coefficient; B, unstandardized coefficient; CI, confidence interval; DV, dependent variable; IV, independent variable; SE, standard error for B.

According to the multiple binary logistic regression analyses (with −2 Log likelihood values ranging between 696.585 and 699.971, Cox and Snell R‐Squared values ranging from 0.017 to 0.023, and Nagelkerke R‐Squared values ranging from 0.023 to 0.031), sex or age were not associated the diagnosis of BDD based on the sum of the first three questions (Table 7). Each of the variables IOTN‐AC or IOTN‐ACE would be significant only if the other one was excluded (Table 7).

**Table 7:** The results of the multiple binary logistic regression analysis for predictors of BDD diagnosis based on BDD‐3 (the sum of the first three questions).

	Predictor	*B*	SE	*p*	OR	95% CI	
Model 1	Sex	−0.223	0.226	0.325	0.800	0.514	1.246
	Age	0.014	0.017	0.410	1.014	0.981	1.047
	Education	−0.017	0.156	0.912	0.983	0.725	1.333
	Marital status	−0.442	0.304	0.146	0.643	0.354	1.166
	Rhinoplasty	0.515	0.722	0.475	1.674	0.407	6.895
	Time	0.003	0.076	0.966	1.003	0.864	1.164
	IOTN‐AC	0.081	0.044	0.066	1.084	0.995	1.182
	IOTN‐ACE	0.056	0.047	0.235	1.058	0.964	1.160
	Constant	−1.044	0.398	0.009	0.352		
Model 2	Sex	−0.214	0.225	0.342	0.807	0.519	1.256
	Age	0.014	0.017	0.397	1.014	0.982	1.048
	Education	−0.026	0.155	0.869	0.975	0.720	1.320
	Marital status	−0.473	0.302	0.117	0.623	0.345	1.126
	Rhinoplasty	0.495	0.722	0.493	1.641	0.398	6.756
	Time	−0.028	0.071	0.697	0.973	0.846	1.119
	IOTN‐AC	0.103	0.040	0.010	1.108	1.025	1.198
	Constant	−0.851	0.362	0.019	0.427		
Model 3	Sex	−0.236	0.225	0.296	0.790	0.508	1.229
	Age	0.012	0.017	0.477	1.012	0.980	1.045
	Education	−0.024	0.155	0.875	0.976	0.720	1.323
	Marital status	−0.402	0.302	0.183	0.669	0.371	1.209
	Rhinoplasty	0.505	0.721	0.484	1.657	0.403	6.811
	Time	−0.001	0.076	0.985	0.999	0.861	1.158
	IOTN‐ACE	0.092	0.043	0.031	1.097	1.009	1.193
	Constant	−0.851	0.382	0.026	0.427		

Abbreviations: B, unstandardized coefficient; CI, confidence interval; OR, odds ratio; SE, standard error for B.

## 4 Discussion

Overall, the possibility of a weak, but statistically significant, correlation between BDD and IOTN‐AC or IOTN‐ACE was intriguing. This could mean that milder orthodontic cases are slightly more likely to result in BDD, or that patients with BDD frequently visit orthodontic clinics even when their treatment needs are less severe [22]. This was consistent with an earlier study that found IOTN‐DHC to be inversely correlated with BDD [22]. As a consequence, similar to a recent paper [22], we also do not support the assertion made by Philips [32] that the answers to the first three questions accurately represent the whole questionnaire, particularly when it comes to binary diagnoses (which were employed in many previous studies). As a result, using the entire questionnaire or creating more thorough ones could be preferable. With the results of lower IOTN‐AC/ACE with longer treatment duration and no significant difference in BDD‐All/‐3 with it, we described the possibility that the orthodontic treatment might decrease IOTN‐AC/ACE and that individuals may have lighter treatment demands (IOTN‐AC/ACE) but more severe OCD or BDD features. However, these results could be interpreted differently. The evidence is weak because this is not a longitudinal study; we have now conducted additional studies to address this concern. Moreover, because any surgical or invasive procedures to change appearance, including orthodontic treatment, generally might not improve the symptoms of BDD or might even exacerbate them, such treatments are not recommended [33]. As the interpretation of these results, even if IOTN‐AC/ACE can be improved by orthodontic treatment, BDD traits might not be; of course, this is a question to be answered in our future article on this topic. But if this assumption holds, when a high tendency of BDD is detected, this should be considered before the initiation of orthodontic treatment. The correlations between BDD and IOTN‐AC/ACE were very weak; however, BDD traits should be carefully assessed when IOTN‐AC/ACE are high to prevent excessive invasive treatment.

BDD prevalence in orthodontic/orthognathic patients is non‐trivial (about 6% or 10% calculated in different systematic reviews), reinforcing the importance of systematic psychological screening before treatment. This provides an external benchmark and underscores the clinical value of screening [34, 35]. Based on all 12 criteria, we estimated that 15% of orthodontic patients may have BDD, which was much higher than a previous study on orthodontic patients [22] and somewhat higher than the general population [19] but comparable to cosmetic patients [8, 19, 20]. The prevalence of BDD rose to 39% based on the three questions of the BDD questionnaire, which were utilized in all three of the earlier investigations on BDD in orthodontics. Compared to the reported prevalences of 5.2%–17% in orthodontic patients of other studies [8, 13, 17, 22, 36], this was much higher. It is quite challenging to comprehend the cause of the debate in the absence of more orthodontic patient research. However, two of these studies had relatively small samples, which may have an impact on the outcomes for methodological reasons. Considering BDD‐3 presented a higher ratio of BDD‐positive patients compared to BDD‐All, it may have the potential for overestimated detection of BDD. Therefore, BDD‐All would be more reasonable for the binary diagnosis of BDD. Additionally, socioeconomic factors from other studies may be important when evaluating oneself [3]. Statistical analyses may also be a significant element. Bivariable statistics were employed in nearly all earlier research, and the majority of these investigations divided the patients into groups that were BDD‐positive and BDD‐negative. The BDD scores in this research were unaffected by age. Whether aging may affect self‐evaluation [3] or not [37] is up for debate. Age may not have an impact on BDD in orthodontic patients, according to two earlier research [8, 22], while another study found the contrary [17]. Numerous factors, such varying age ranges or socioeconomic conditions, might be to blame for this [22]. According to the current study, BDD was more common among women. This outcome was consistent with the majority of earlier research [17, 22, 38, 39, 40, 41, 42]. However, some research reported no difference between the sexes [43], while others indicated a higher frequency in males including a study in orthodontic patients [8, 23, 36]. The severity of BDD, eligibility requirements, and referral bias may be responsible for some of these discrepancies [13, 41, 44]. Additionally, as this study shown, women and men often vary primarily in some, but not all, aspects of the obsessive‐compulsive domain [22]. Contrary to a previous study [22], the current investigation did not reveal a greater prevalence of BDD in married patients than in single patients, which was in line with nearly all other publications in this area [8, 13, 17, 45]. This study failed to find associations between esthetic treatments and BDD; of course, the number of such patients was too small to allow reliable results in this regard. Although the time past the beginning of treatment was not associated with BDD, it was associated with IOTN‐AC and IOTN‐ACE; obviously, it can be expected that with treatment, IOTN‐AC/ACE scores reduce to better values.

This study might be limited by some factors. The cross‐sectional design of the study was a limitation. Moreover, the lack of objective malocclusion, that is, the dental health component of IOTN was another limitation. In addition, there may be residual confounders like anxiety and stress, not addressed in this study. The observed psychological traits can be limited to the geographical regions, socioeconomic statuses, and other factors. Besides, orthodontic norms may differ between various ethnicities. These limit the results to this particular race and ethnic group. Besides, the number of cases with esthetic treatment was small. It should be noted that the BDD questionnaire in use cannot replace a thorough clinical examination and diagnosis. Although we mentioned terminologies like BDD diagnosis based on the literature, none of these diagnoses were made clinically by a psychiatrist, after several sessions of evaluation. Still, a high score on the BDD questionnaire can imply a high possibility of a positive BDD diagnosis if examined clinically.

## 5 Conclusions

Within the limitations of this study, and also emphasizing that the screening questionnaires used cannot replace a rigorous clinical judgement by an experienced psychiatrist, the following may be concluded: According to the 3‐item (BDD‐3) and 12‐item (BDD‐All) questionnaires, the prevalence of BDD in orthodontic patients was 39.3% and 15.1%, respectively. The majority of BDD‐positive patients had mild BDD. Although the BDD‐3 score strongly correlated with BDD‐All scores, their binary diagnoses were not correlated strongly. Hence, given the potential for overestimation of BDD detection by BDD‐3, BDD‐All may be more preferable for binary diagnosis. According to IOTN‐AC and IOTN‐ACE, the majority of orthodontic patients had mild to moderate normative treatment needs. Age negatively correlated with IOTN‐AC/ACE, and higher IOTN‐ACE scores were observed in single patients. The time passed since the beginning of treatment is associated with the decline of IOTN‐AC or ‐ACE scores. Further longitudinal research is needed to elucidate this.

Female sex and IOTN‐AC predict higher scores in BDD‐All, as well as IOTN‐AC predicts a binary diagnosis based on BDD‐All. Moreover, IOTN‐AC predicts higher scores of BDD‐3, and IOTN‐AC/ACE predicts a binary diagnosis using BDD‐3. Since some individuals may have lighter treatment demands but more severe OCD or BDD features, this should be taken into account while making treatment plans.

## Author Contributions

Mehrnaz Moradinezhad proposed the idea, searched the literature, conceived the study, designed the study, supervised the thesis, drafted the article and critically reviewed the article. Ozra Niknam conceived the study, supervised the thesis, drafted the article and critically reviewed the article. Majid Aryannejad collected the data. Mahsa Shishesaz searched the literature, collected the data, wrote the thesis, and critically contributed to the article. All authors viewed the final version of the article and agreed to submit it to this journal. Asal Fetrati contributed to literature search, interpretation of results, and drafting the manuscript. Vahid Rakhshan analyzed the data, drafted the manuscript, created the tables and figures.

## Funding

The authors have nothing to report.

## Ethics Statement

The protocol and its ethics were approved by the Research Ethics Committee of Ahvaz Jundishapur University of Medical Sciences, Ahvaz, Iran (Ethics Code: IR. AJUMS. REC.1402.358). All methods were performed in accordance with the relevant guidelines and regulations (including the Declaration of Helsinki); all experimental protocols were approved by the Institutional Review Board of Ahvaz Jundishapur University of Medical Sciences, Ahvaz, Iran (ethics code: IR.AJUMS.REC.1402.358).

## Consent

The study's goals and methodology were described to all participants (and in the case of underaged children, to their parents). Patients and their parents were guaranteed about the confidentiality of their information. Finally, all patients and/or their parents (in the case of children) signed written informed consent forms.

## Conflicts of Interest

The authors declare no conflicts of interest.

## Transparency Statement

The corresponding author, Mahsa Shishesaz, affirms that this manuscript is an honest, accurate, and transparent account of the study being reported; that no important aspects of the study have been omitted; and that any discrepancies from the study as planned (and, if relevant, registered) have been explained.

## Supporting information

Supporting File:

## Data Availability

The data are available upon request from the corresponding author (M.S.).
