# Supplementary material for: Links Between Body Dysmorphic Disorder (BDD) With Aesthetic Components of Orthodontic Treatment Need Assessed by Orthodontic Patients or Orthodontists (IOTN‐AC, IOTN‐ACE) and Also With Its Risk Factors: An Epidemiological Cross‐Sectional Study
Source: Health Sci Rep. 2026 May 8;9(5):e71956. doi: 10.1002/hsr2.71956 (PMC13156402; doi:10.1002/hsr2.71956)
Supplement: Supplementary file 1 — Supporting File: [file HSR2-9-e71956-s001.docx]

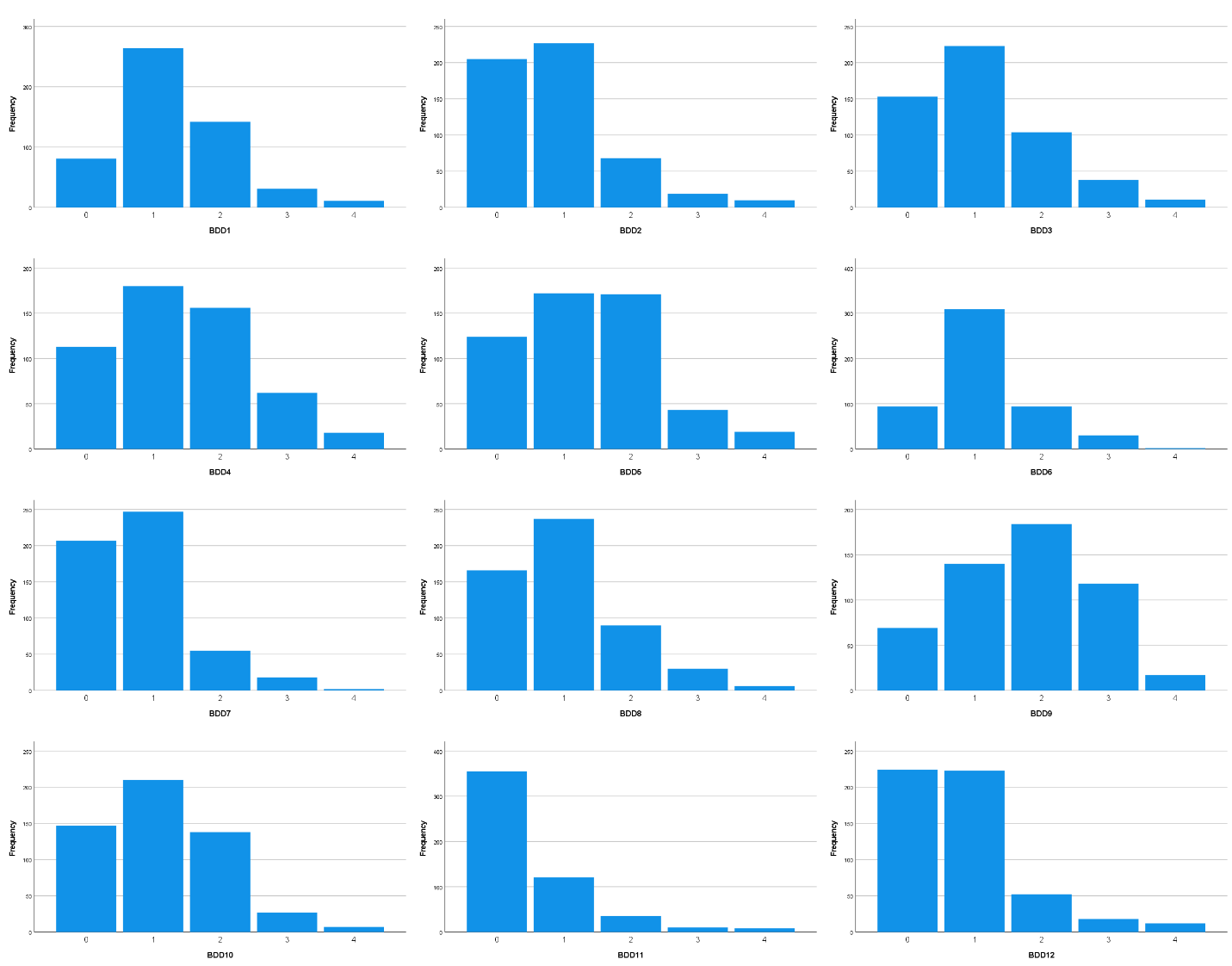
**Supplementary Figure 1.** Distributions of answers to each of 12 BDD questions (n = 529). The distributions of milder cases are greater than severer cases.
